# Optimization of a Time-of-Arrival-Ridge Estimation Iterative Model for Ultra-Wideband Positioning in a Long Linear Area

**DOI:** 10.3390/s25072229

**Published:** 2025-04-02

**Authors:** Mengqian Li, Mingduo Li, Jinhua Wang, Aoze Duan, Haotian Sun, Qinggang Meng

**Affiliations:** 1 College of Mining Engineering, North China University of Science and Technology, Tangshan 063210, China; limengqian9163@ncst.edu.cn (M.L.); daz0802@163.com (A.D.); sht1178521440@163.com (H.S.); mengqg@stu.ncst.edu.cn (Q.M.); 2 College of Electrical Engineering, North China University of Science and Technology, Tangshan 063210, China

**Keywords:** UWB indoor positioning, long linear area, ridge estimation, equivalent weight factor

## Abstract

**Highlights:**

**What are the main findings?**

- A high-precision UWB+TOA-RR positioning algorithm is proposed, incorporating Ridge estimation and equivalent weights to improve localization accuracy in long linear areas.
- The TOA-RR model achieves a positioning accuracy of approximately 0.5 m, significantly outperforming conventional TOA-LS and TOA-R models in long linear environments.

**What is the implication of the main finding?**

- This study resolves the challenge of inaccurate UWB localization in long linear areas, such as tunnels and corridors, providing a robust solution for high-precision indoor positioning.
- The proposed TOA-RR algorithm enhances the stability and accuracy of UWB localization, making it suitable for practical applications in challenging environments.

**Abstract:**

Ultra-wideband (UWB) technology is widely used for high-precision indoor positioning due to its adaptability to various environments. However, in long linear areas, such as tunnels or corridors, the near-linear deployment of base stations caused by structural constraints significantly degrades UWB localization accuracy, rendering conventional algorithms ineffective. To address this issue, this study proposes a high-precision UWB+TOA-R positioning algorithm that incorporates Ridge estimation as a constraint condition. The algorithm introduces equivalent weights to refine the iterative computation of Ridge estimation, establishing an iteratively computed TOA-RR solution model. Experiments were conducted in a long linear corridor to compare the performance of three UWB localization models: the TOA-Least Squares (TOA-LS) model, the TOA-Ridge estimation (TOA-R) model, and the proposed TOA-Ridge estimation iterative (TOA-RR) model. The results indicate that the TOA-LS model suffers from significant coordinate distortions due to abnormalities in the inverse matrix of the coefficient matrix, regardless of the initial tag coordinates. The TOA-R model demonstrates improved accuracy and stability, particularly in cases of significant initial deviations, but still exhibits residual errors. In contrast, the TOA-RR model achieves enhanced stability and accuracy, with a positioning error of approximately 0.5 m. This study resolves the challenge of inaccurate UWB localization in long linear areas, providing a robust solution for such environments.

## 1. Introduction

The rapid development of intelligent location services requires high-precision indoor localization technology. At present, the technologies utilized for indoor localization can be classified into the following categories: distance sensing based on ultra-wideband (UWB), Wi-Fi RTT (round-trip time) ranging technology, audio positioning technology, Bluetooth goniometry technology, and visual positioning technology. Among these, UWB localization has a high hardware construction cost and high accuracy. Some results indicate that, in a rectangular area, the positioning accuracy can reach 10 cm if the positioning tag collects signals from 3–4 base stations simultaneously. However, in long linear corridors or tunnels, the positioning tag can only receive 1–2 base station’s signals, and the optimal positioning accuracy is approximately at the sub-meter level [1,2,3].

In order to improve the positioning accuracy and stability of UWB, some scholars have made certain advancements in the deployment of UWB base stations and the methodologies for model solutions. However, there is a scarcity of research dedicated to improving the accuracy of UWB positioning in long linear areas. In a long linear area, such as a tunnel or a corridor, the UWB base station signal transmission is constrained; in general, only two base stations’ signals can be received. And there is a linear correlation between the spatial location of the two base stations. The least squares solving results of UWB positioning revealed that accuracy in these conditions is extremely unstable. Currently, there is a dearth of research exploring the potential for enhancing the precision of UWB localization in this context.

In order to address the issue of the correlation problem of base station coordinates in long linear tunnels, the UWB Ridge estimation solution model is proposed. The ridge parameter estimation method and iterative equations in the solution model are constructed to improve the reliability and stability of the UWB solution results in the long linear area.

### 1.1. Related Work

The UWB positioning system typically comprises three core components: unknown location tags, a known location base station, and a data processing terminal. The commonly used positioning method is to estimate the distance or angle from the pending tags to multiple base stations by utilizing the magnitude of the intensity value of the collected signals and subsequently calculating the coordinates of the tags. In terms of the localization techniques, the most frequently employed methods encompass Angle of Arrival (AOA), Time of Arrival (TOA), Time Difference of Arrival (TDOA), and Time of Flight (TOF) [4,5,6,7,8]. The commonly utilized algorithms include Taylor’s series algorithm, the least squares algorithm, Chan’s algorithm, and the Gauss–Newton algorithm, among others.

As documented in the references, these positioning methods and algorithms have achieved a certain accuracy in rectangular indoor positioning [9,10,11,12]. However, there are still several challenges persist, including the optimization of base station layout and the instability of calculation coordinate accuracy. Numerous scholars have conducted extensive research aimed at enhancing positioning accuracy through the optimization of UWB equipment layout and the refinement of model solution methodologies.

(1) In the domain of optimizing UWB equipment layout schemes and exploring UWB-based combination positioning methods, several scholars have conducted studies and achieved more favorable outcomes.

Yan et al. proposed a GNSS/UWB fusion algorithm for indoor and outdoor crossing areas. Helmert variance component was used to estimate the uncertainty of UWB and GPS measurement values, and the weight of observations was re-adjusted for the fusion solution, thus improving the comprehensive positioning accuracy [13]. Liu Xin et al. designed an integrated positioning system based on UWB positioning technology and a TOF/PDOA joint algorithm. The experimental tests demonstrated good positioning accuracy, with static positioning errors less than 0.3 m [14]. Ali, Rashid fused IMU PDR and UWB measurements using the Extended Kalman Filter (EKF) algorithm, a powerful tool for nonlinear state estimation, to provide a general solution for indoor positioning, enhancing the accuracy of indoor positioning [15]. Si Minghao et al. proposed a hybrid indoor altimeter based on a barometer and UWB sensor to increase the positioning accuracy by measuring the current height information through the barometer and using a UWB sensor for time synchronization and data transmission [16]. Chen, Z.J. et al. constructed a tightly coupled UWB/LiDAR-SLAM positioning system, using UWB line-of-sight measurements and LiDAR-SLAM positioning information to improve the inaccuracy of ultra-wideband ranging and localization under non-line-of-sight conditions [17]. Wu Junkang et al. studied the multipath problem of UWB signals and found that, in NLOS environments, the actual positioning accuracy can be improved by employing the double-sided two-way ranging method based on TOA [18]. Wang Guijie et al. proposed that by positioning UWB base stations at different elevations, the error in tag coordinates is controlled within a certain range using the least square solution, with the error tending to be stable [19]. Harris Perakis et al. investigated the integrated positioning technology based on UWB and Wi-Fi RTT, and proposed a distributed collaborative positioning algorithm combining the Extended Kalman Filter (EKF) and the Split Covariance Intersection Filter (SCIF) [20].

(2) Numerous scholars carried out optimizations of UWB solution models, yielding positive application outcomes.

Zhu Chunhua et al. developed a novel TOF and TDOA joint positioning approach by incorporating TOF ranging and a weighted centroid localization algorithm. This novel algorithm was capable of delivering greater positioning accuracy and an expanded positioning range, even in the presence of significant noise [21]. Kun Zhao et al. scrutinized the sources of positioning errors, enhanced the time synchronization algorithm, and further optimized the time synchronization performance utilizing the labels at known locations, thus optimizing the TDOA measurement outcomes [22]. Qian Jianhang employed a TDOA model and weighted least squares mathematical algorithm to mitigate the significant impact of non-line-of-sight (NLOS) errors on UWB indoor positioning, and the accuracy in both X and Y directions saw an approximate 50% improvement when utilizing the weighted least squares algorithm [23]. Liu Gaohui et al. introduced a recursive hybrid TDOA/FDOA positioning method that integrates the weighted least squares and genetic algorithm. The simulation experiments showed that the positioning accuracy of this method could be enhanced and mitigated the effects of substantial observational errors [24]. Poulose, A et al. proposed a long short-term memory (LSTM) network model to receive distance measurements from the TOA-distance model of the UWB system for user position prediction [25].

(3) Furthermore, the research into UWB technology has yielded promising results in the field of long linear underground tunnel localization.

To enhance the initial positioning accuracy of ultra-wideband, a computation method that integrates the Taylor series and the Bacteria Foraging Optimization Algorithm (BFOA-Taylor) proposed a computation method by Si, L. This method employs the ultra-wideband confidence factor to dynamically adjust the fusion weights of ultra-wideband information, thereby mitigating the influence of non-line-of-sight (NLOS) errors and achieved accurate positioning [26]. To enhance the comprehensive positioning accuracy of unmanned autonomous vehicles in coal mines, Cui, Y.M. developed an 18-dimensional model of an odometer-aided Inertial Navigation System (INS) and a positioning model of an EKF for ultra-wideband (UWB) systems both based on the vehicle kinematics equation [27]. Cui, Y.M. et al. developed a multi-sensor fusion positioning system designed to enhance the accuracy of positioning in long and narrow Global Positioning Systems (GPSs) denied for underground coal mine roadways. This system, leveraging the vibration characteristics of underground mobile devices, integrated Inertial Navigation System (INS), odometer, and ultra-wideband (UWB) technologies via an EKF and an Unscented Kalman Filter (UKF) [28].

In summary, the majority of studies on UWB positioning methods have focused on integrating UWB with other autonomous positioning technologies or enhancing positioning accuracy through the optimization of UWB models. However, there is a scarcity of studies focusing on the optimization of the constraint rules within model algorithms. Especially in linear areas of tunnels or corridors, the signal transmission of UWB base stations is limited or susceptible to interference from the external environment. Most studies on linear areas focus on improving positioning accuracy by incorporating auxiliary measurement systems, but in the case of linear correlations between the spatial positions of base stations, there is a dearth of research on the application of TOA-constrained model optimization to enhance UWB positioning accuracy.

### 1.2. Motivation and Contribution

To address the correlation issue of base station coordinates in long linear areas, which may result in inaccuracies in positioning systems, this study proposes a UWB+TOA positioning model that incorporates Ridge estimation as a constraint. On this basis, a TOA-RR model is constructed, which searches for the optimal solution by optimizing weight values to adjust the iterative computation of Ridge estimation. This approach significantly enhances the reliability and stability of the UWB positioning results in long linear areas.

### 1.3. Outline of the Paper

In this paper, Section 1 provides an overview of the research background and the primary innovative contributions. Section 2 reviews the existing literature on the existing autonomous localization methods. Section 3 constructs the mathematical model for the UWB positioning model, utilizing Time-of-Arrival (TOA) and Ridge estimation methodologies, and introduces the equivalent weights to adjust the iterative calculation results of the Ridge estimation. Section 4 outlines the test scheme, test site, test equipment, test process, and evaluation metrics, and analyzes the test results. Section 5 presents the conclusion.

## 2. Optimization of UWB Positioning Calculation Models

### 2.1. TOA-LS (Least Squares Model)

**Figure 1:**
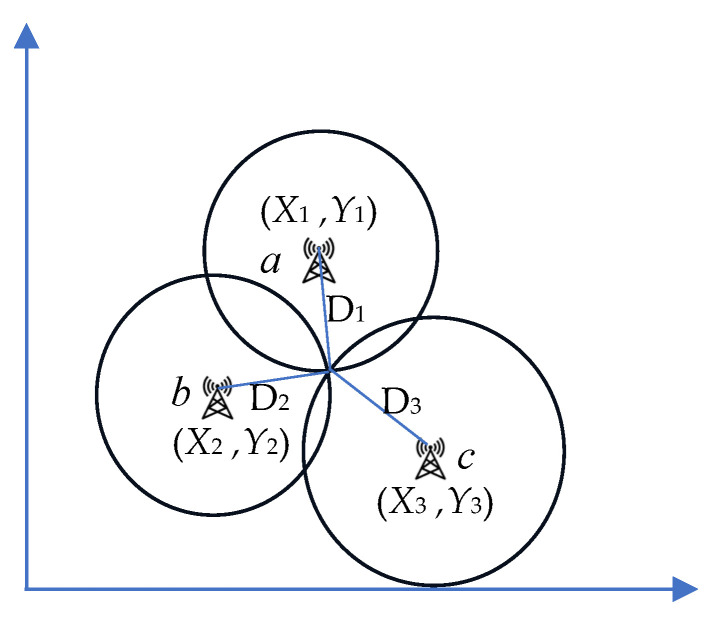
TOA positioning principle.

The principle of TOA positioning is to estimate the delay from the pending tag to the reference base station, so as to obtain the distance between them, and the TOF (Time-of-Flight) algorithm is used to calculate the distance between the tag to be located and the reference base station, as shown in Figure 1.

TOA positioning formulates the observation equation according to the time when the monitoring positioning signal arrives at the base station, and it leverages the clock skew between the tags to be located and the base station to resolve the unknown coordinates. Once the distance from two or more pending tags to the base station is obtained, the coordinates of these tags can be accurately determined. Assume that ${\hat{D}}_{ji}$ represents the adjusted value of the distance from pending tag *i* to reference base station j (*j* = *a*, *b*, *c*, ……), where the coordinates of the base station are known. The equation is

(1) $${\hat{D}}_{ji}=D_{ji}+v_{i}=\sqrt{{\left(X_{j}-{\hat{X}}_{i}\right)}^{2}+{\left(Y_{j}-{\hat{Y}}_{i}\right)}^{2}}$$

Let $X_{i}^{0},Y_{i}^{0}$ be the initial values of the coordinates of the pending tag, set as ${\hat{X}}_{i}=X_{i}^{0}+{\hat{x}}_{i}$, ${\hat{Y}}_{i}=Y_{i}^{0}+{\hat{y}}_{i}$.

Employing the Taylor series expansion and neglecting higher-order terms beyond quadratic, the error equation for the *i*-th measured distance is derived as follows:

(2) $$v_{i}=-\frac{\Delta X_{ji}^{0}}{D_{ji}^{0}}{\hat{x}}_{i}-\frac{\Delta Y_{ji}^{0}}{D_{ji}^{0}}{\hat{y}}_{i}-l_{i}$$

where $\Delta X_{ji}^{0}=X_{j}^{0}-X_{i}^{0},\Delta Y_{ji}^{0}=Y_{j}^{0}-Y_{i}^{0}$, $D_{ji}^{0}=\sqrt{{\left(X_{j}^{0}-X_{i}^{0}\right)}^{2}+{\left(Y_{j}^{0}-Y_{i}^{0}\right)}^{2}}$, $l_{i}=D_{ji}-D_{ji}^{0}$.

Therefore, when the coordinates of three base stations a, b, and c are known and the coordinate of pending tag *i* is $\left({\hat{X}}_{i},{\hat{Y}}_{i}\right)$, the data processing error model is:

(3) V=BX−L

where $B=\left[\begin{array}{cc} \frac{\Delta X_{ia}^{0}}{D_{ia}^{0}} & \frac{\Delta Y_{ia}^{0}}{D_{ia}^{0}}\\ \frac{\Delta X_{ib}^{0}}{D_{ib}^{0}} & \frac{\Delta Y_{ib}^{0}}{D_{ib}^{0}}\\ \frac{\Delta X_{ic}^{0}}{D_{ic}^{0}} & \frac{\Delta Y_{ic}^{0}}{D_{ic}^{0}} \end{array}\right],X=\left[\begin{array}{c} X_{i}\\ Y_{i} \end{array}\right]=\left[\begin{array}{c} X_{i}^{0}+\delta x_{i}\\ Y_{i}^{0}+\delta y_{i} \end{array}\right],L=\left[\begin{array}{c} l_{1}\\ l_{2}\\ l_{3} \end{array}\right]$

In this equation, $\delta x_{i}$ and $\delta y_{i}$ represent the corrections to the initial coordinates of the tag in the *X* and *Y* directions.

In accordance with the least squares (LS) method, the constraint law states that

(4) $$V^{T}PV=min$$

The model currently in use assumes that UWB multi-baseline ranging constitutes equal-precision observations, hence the covariance matrix p is an identity matrix.

(5) $$V^{T}V=min$$

The coordinates of the pending tag can be estimated using the following procedure:

(6) $$X_{LS}={\left(B^{T}B\right)}^{-1}B^{T}L$$

Equation (6) is more effective for rectangular or square interiors, where multiple measurement baselines originate from different known coordinates of the base stations, there is no linear correlation between these observations, and the accuracy of the solution typically achieves meter-level precision. When the base station is laid out in a corridor or tunnel, the long and narrow structure, typically 2–3 m in width and tens of meters in length, is similar to beads strung on a line. This configuration leads to a strong correlation between the coordinates of the base stations. If the *LS* estimation defined in Equation (6) is used for the calculation, $N=B^{T}B$ ranks deficient or close to singular, and the result of $X_{LS}$ is seriously distorted. Some of the test results indicate that the margin of error may extend to several meters.

### 2.2. TOA-R (Ridge Estimation Model)

The Ridge estimation (RE), proposed by Hoerl and Kennard in 1970, represents a biased estimation technique [29]. It is particularly applicable for solving equations where the structure of the known observations is irrational and the normal equations are rank deficient or nearly singular. The Ridge estimation (RE) constraint method introduces a parameter, *k*, known as the ridge parameter, to the normal equations by augmenting $B^{T}B$ with a diagonal matrix, *kI*(*k* > 0). Upon selecting an appropriate value for the ridge parameter, *k*, eigenvalues $\lambda_{i}+k$ of $B^{T}B+kI$ are not close to zero,

(7) $${\hat{X}}_{RE}\left(k\right)={\left(N+kI\right)}^{-1}B^{T}L$$

Here, *k* represents the ridge parameter, defined as an arbitrary positive constant: $N=B^{T}B$. When different values of *k* are chosen, different Ridge estimates are obtained. Ridge estimation mitigates the complex multicollinearity among the original coefficients, resulting in a Mean Squared Error (MSE) that is lower than that of the least squares (LS) estimation. This improves the least squares estimation to some extent and bolsters the model’s accuracy.

The parameters of RE are typically calculated based on the least squares (LS) results, employing the double-*h* formula method, as detailed below:

(8) $$k=\frac{h_{1}{\hat{\sigma }}_{0}^{2}}{{\hat{X}}_{LS}^{T}G{\hat{X}}_{LS}+h_{2}{\hat{\sigma }}_{0}^{2}}$$

Generally, G = *I*, $h_{1}=t$, and $h_{2}=0$, where *t* represents the number of parameters in the mathematical model.

(9) $$k=\frac{t{\hat{\sigma }}_{0}^{2}}{{\hat{X}}_{LS}^{T}{\hat{X}}_{LS}}$$

The formula for the unit weighted error, denoted as $\;\sigma_{0}$, is presented by $\sigma_{0}=\pm \sqrt{\frac{V^{T}V}{n-t}}$. Here, *n* represents the number of observed distances from the tag to the base station. The variable *t* signifies both the number of unknown parameters in the model and the number of necessary observation parameters. In this paper, *t* is set to 2 when determining the point position coordinates.

### 2.3. TOA-RR (Ridge Estimation Iterative Model)

Equation (2) represents the Taylor series expansion of the TOA positioning model, with quadratic and higher-order terms omitted. This corresponds to the linear form when derived at a certain initial value. If the initial value is inaccurately estimated or deviates significantly from the true value, the error in the linearized model of Equation (2) can become substantial, potentially creating significant distortion in the final solved coordinates. To enhance the accuracy of UWB-TOA-R position coordinates, the weights can be optimally adjusted under the Ridge estimation constraint, thereby minimizing the calculation error due to the strong correlation among base stations as much as possible. Consequently, an iterative convergence method can be employed to determine the optimal value. An equivalent weight is added to Equation (7), which is calculated as the product of the previous weight and the equivalent weight factor.

(10) $${\hat{X}}_{RR}\left(i\right)={\left(B^{T}{\bar{P}}^{\left(i\right)}B+k^{\left(i\right)}I\right)}^{-1}B^{T}{\bar{P}}^{\left(i\right)}L$$

(11) $${\bar{P}}^{\left(i\right)}=P^{\left(i\right)}W^{\left(i\right)}$$

(12) $$W^{\left(i\right)}=\left[\begin{array}{c} w_{1}^{\left(i\right)}\\ w_{2}^{\left(i\right)}\\ \vdots \end{array}\right]=\left\{\begin{array}{c} 1\;\left|v_{i}^{\left(i-1\right)}\right|<\theta \\ \frac{v_{i}^{\left(i-1\right)}}{\theta }\;\left|v_{i}^{\left(i-1\right)}\right|\geq \theta \end{array}\right.$$

Here, ${\bar{P}}^{\left(i\right)}$ represents the equivalent weight calculated during the *i*-th iteration, and $W^{\left(i\right)}$ is the equivalent weight factor associated with the observations. $v_{i}^{\left(i-1\right)}$ denotes the corrections to the observations following the (*i* − 1)th iteration. The model’s limit error is presented by θ=kσ, where *k* typically ranges from 0.5 to 2.

The TOA-RR iterative model encompasses the following eight steps.

Step1: Calculate the approximate coordinates of the tag $X_{i}^{0},Y_{i}^{0}$;

Step2: Solve the TOA based on the least square principle to obtain the initial estimate of the parameter $\hat{X}$ and the residual *V*;

(13) $${\hat{X}}_{LS}={\left(B^{T}B\right)}^{-1}B^{T}L$$

(14) $$V^{\left(1\right)}=BX_{LS}-L$$

(15) $${\hat{\sigma }}_{0}^{2}=\frac{V^{T}V}{n-t}$$

Step 3: Calculate the ridge parameters using the subsequent equation:

(16) $$k^{\left(1\right)}=\frac{t{\hat{\sigma }}_{0}^{2}}{{\hat{X}}_{LS}^{T}{\hat{X}}_{LS}}$$

Step 4: A new weight factor $w_{i}$ is calculated for each observation based on the magnitude of the last calculated correction. According to the formula ${\bar{p}}_{i}=p_{i}w_{i}$, the new equivalent weight ${\bar{P}}^{\left(1\right)}$ is constructed.

(17) $$w_{i}^{\left(1\right)}=\left\{\begin{array}{c} 1\;\left|v_{i}^{\left(1\right)}\right|<\theta \\ \frac{v_{i}^{\left(1\right)}}{\theta }\;\;\;\;\left|v_{i}^{\left(1\right)}\right|\geq \theta \end{array}\right.$$

Step 5: Leveraging the insights derived from Step 2, the second estimate of the parameter and the residual *V* are computed utilizing the ridge parameter solution algorithm.

(18) $${\hat{X}}^{\left(2\right)}={\left(B^{T}{\bar{P}}^{\left(1\right)}B+k^{\left(1\right)}I\right)}^{-1}B^{T}{\bar{P}}^{\left(1\right)}L$$

(19) $$V^{\left(2\right)}=B{\hat{X}}^{\left(2\right)}-L$$

Step 6: The new ridge parameter is *c*, then calculated using the subsequent equation.

(20) $$k^{\left(2\right)}=\frac{t{\hat{\sigma }}_{0}^{2}}{X^{\left(2\right)T}{\hat{X}}^{\left(2\right)}}$$

Step 7: Construct a new equivalent weight ${\bar{P}}^{\left(2\right)}$ from $V^{\left(2\right)}$, then solve the algorithmic equation from Step 5 once more. Subsequently, recalculate the ridge parameters as per Step 6.

(21) $$w_{i}^{\left(2\right)}=\left\{\begin{array}{c} 1\left|v_{i}^{\left(2\right)}\right|<\theta \\ \frac{v_{i}^{\left(2\right)}}{\theta }\;\;\left|v_{i}^{\left(2\right)}\right|\geq \theta \end{array}\right.$$

Step 8: Determine the coordinate difference between the *k* th and the *k* − 1th calculation results. Terminate the operation when the difference is less than 0.001 m or the number of iterations reaches 500. The final result is:

(22) $${\hat{X}}^{\left(k\right)}={\left(B^{T}{\bar{P}}^{\left(k-1\right)}B+k^{\left(k-1\right)}I\right)}^{-1}B^{T}{\bar{P}}^{\left(k-1\right)}L$$

(23) $$\;V^{\left(K\right)}=B{\hat{X}}^{\left(k-1\right)}-L$$

## 3. Test Scheme

### 3.1. Test Site and Equipment

**Figure 2:**
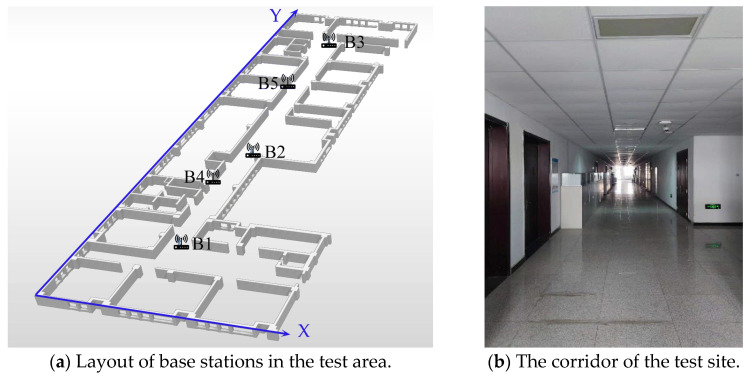
Test site UWB base station layout.

Position the UWB positioning device in a corridor of a building with a width of 3.6 m and a length of 68 m. The origin point of the UWB positioning device is located at the southwest corner, with the base stations linearly arranged along the ceiling of the corridor. The coordinates of these arranged points are detailed in Table 1 and the layout of the UWB base stations is depicted in Figure 2. In our experiment, base stations were alternately positioned along the *x*-axis with a spacing of 1.7 m to prevent ill-conditioned matrices, which can affect the positioning accuracy. This staggered arrangement ensures a more robust multilateration process. In the first instance, the coordinates of the base stations were measured with a high-precision total station theodolite, specifically the NTS-332R10M model, which is capable of achieving measurements with a precision of ±(2 mm + 2 × 10^−6*D*^), where D is the distance in meters. This level of precision is crucial for the accuracy of our TOA-based positioning algorithm.

The base station, which is a U-BASE310 model, should be positioned as indicated in Figure 2 and is responsible for receiving UWB signals emitted by the tags and transmitting the positioning information to the positioning information platform via the network transmission layer. Additionally, base stations transmit synchronization signals to each other to ensure that each positioning base station has a unified clock, thereby obtaining the time difference of the signal’s arrival at different base stations. The operating frequency band of the base station is in the range of 3.1 GHz to 7.0 GHz, with an operating range of 35 to 100 m. The tag operates within the frequency band from 3.1 GHz to 7.0 GHz, and the positioning frequency range is from 0.1 Hz to 50 Hz.

The experiment requires participants to wear the U-TAG 220 model tag and maintain a consistent height difference between the tag and the base station as they move along the corridor.

Th ebase station of U-BASE310 and tag of U-TAG 220 were manufactured by Zhengzhou Lianrui Electronic Technology Co., Ltd., in China (Shenzhen, China). More details can be found at http://www.locarismeta.com (accessed on 30 March 2024).

**Figure 3:**
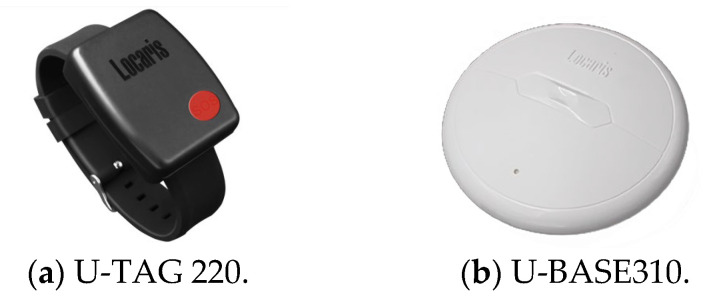
Test equipment.

During the collection of the UWB positioning data, Experimenter A was tasked with carrying the tags. Approximately 100 data points were accumulated for each UWB positioning data collection point. The positioning device operated at a sampling frequency of 10 Hz, indicating a collection rate of 10 data points per second. To ensure an adequate number of samples, the tag must be stationed at the designated sampling point for at least one minute. Electromagnetic wave ranging was employed to gather the coordinates of the test points. See Figure 3.

### 3.2. Data Acquisition and Preprocessing

(1) Distance measurement between the base station and the tag

To enhance ranging accuracy, data acquisition employs the combined measurement method of Time of Flight (TOF) and Time of Arrival (TOA) for the signal. The TOF-based algorithms calculate the distance between the tag and the base station by measuring the round-trip flight time of the signal between them. The tag and the base station are asynchronous transceivers, so the TOF algorithm does not rely on clock synchronization.

**Figure 4:**
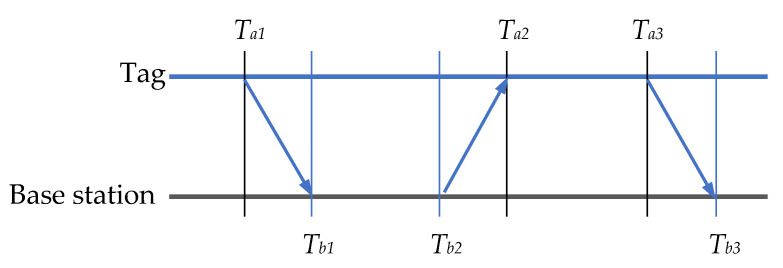
Signal transmission mode between the tag and base station.

In this experiment, we employed the three-message bidirectional ranging method to determine $T_{prop}$. The mode of signal transmission between the tag and base station is depicted in Figure 4. In the three-message two-way ranging method, the tag and the base station interact three times through a wireless transceiver.

The tag initiates the first ranging message and records the signal transmission time $T_{a1}$. The base station receives the first ranging message and records the signal arrival time,$T_{b1}$. Subsequently, the base station sends the second ranging message to the tag, and the tag receives the second ranging message, recording the signal transmission time $T_{b2}$ and arrival time $T_{a2}$. Then, the tag sends the third ranging message to the base station, and the base station receives the third ranging message, recording the transmission time $T_{a3}$ and arrival time $T_{b3}$. Based on these timestamps, the propagation time $T_{prop}$ between the tag and the base station can be calculated using Equation (24).

(24) $$T_{prop}=\frac{\left[\left(T_{a2}-T_{a1}\right)-\left(T_{b2}-T_{b1}\right)\right]+\left[\left(T_{b3}-T_{b2}\right)-\left(T_{a3}-T_{a2}\right)\right]}{4}$$

The distance between the tag and the base station is obtained by multiplying the propagation time $T_{prop}$ and the speed of light, C. This method is compensated by an inverse measurement from reducing the effect of the clock offset and improving the positioning accuracy.

(2) Estimation of the approximate coordinates of the pending tags

Model solving and accuracy validation require initial coordinates. In this study, three types of initial coordinates are obtained: Ground truth coordinates, trilateration coordinates, and refined trilateration coordinates.

Ground truth coordinates are measured using the same total station (NTS-332R10M). The trilateration coordinates are calculated using the trilateration method. The refined trilateration coordinates are obtained by applying a global translation correction to the trilateration coordinates based on the real coordinates. The formulas and computational process for trilateration are described below.

**Figure 5:**
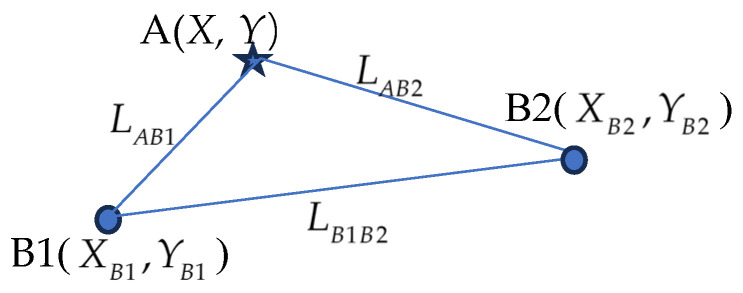
Triangle of points to be measured.

Tag A to be measured forms a triangle with any two base stations, B1 and B2, as shown in Figure 5. Taking the triangle formed by tag A and base stations B1 and B2 as an example, the computational process is described below.

Step 1: Calculate the distance, $L_{B1B2}$, between base stations B1 and B2.

(25) $$L_{B1B2}=\sqrt{{\left(X_{B1}-X_{B2}\right)}^{2}+{\left(Y_{B1}-Y_{B2}\right)}^{2}}$$

Step 2: Compute the trigonometric values of angle B2, i.e., cosB2 and sinB2.

(26) $$\cos B2=\frac{L_{B1B2}^{2}+L_{AB2}^{2}-L_{AB1}^{2}}{2L_{B1B2}L_{AB2}},\;\sin B2=\sqrt{1-\cos^{2}B2}$$

Step 3: Calculate the initial coordinates.

(27) $$X_{i}^{0}=X_{B2}+\Delta X=X_{B2}+L_{AB2}\cdot \cos B2’Y_{i}^{0}=Y_{B2}+\Delta Y=Y_{B2}+L_{AB2}\cdot \sin B2$$

The initial coordinates obtained in Step 3 are based on a single triangle calculation. However, when tag A receives signals from more than two base stations, multiple initial coordinates can be calculated. The average of these coordinates is taken as the trilateration position of the tag.

**Figure 6:**
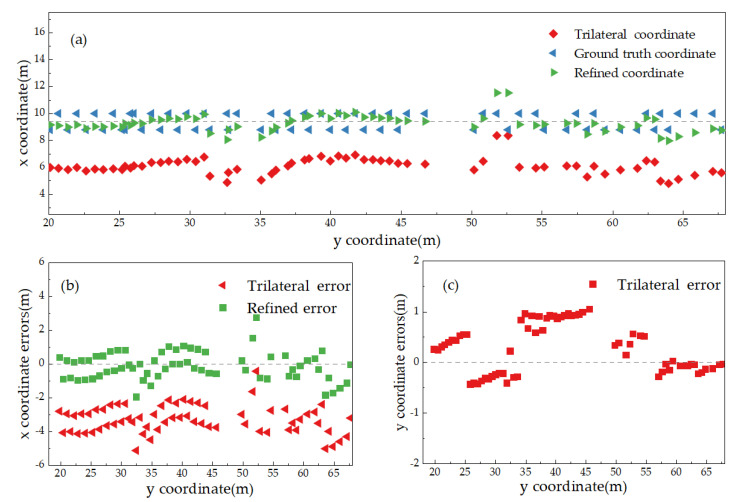
Approximate coordinates of the pending tags: (**a**) measurement points, (**b**) *x*-coordinate errors, (**c**) *y*-coordinate errors.

Figure 6a presents the trajectory diagram of the actual point coordinates, the coordinates calculated using the trilateration method, and the refined trilateration intersection coordinates within the B1–B5 region. From the figure, it is evident that the UWB positioning system experiences signal transmission errors and exhibits poor stability during the ranging process. Consequently, the distance between the tag and the base station, determined based on the Time of Flight, is subject to a substantial error. This, in turn, results in a considerable discrepancy in the initial coordinates of the tag, as determined by the trilateration method. As shown in Figure 6b,c, the trilateration method for coordinate determination shows superior accuracy in determining the *y*-coordinate over the *x*-coordinate. This is primarily due to the corridor’s narrowness, minor discrepancies in the *x*-coordinates of the base stations, and the linear correlation of the data, all of which collectively reduce the precision of the solution.

Given that the trilateration solution’s outcome is displaced by approximately 3 m in the *x*-direction relative to the true coordinates, the *x*-coordinate derived from the trilateration solution is adjusted by 3 m toward the true coordinates to obtain the refined trilateration intersection coordinates.

### 3.3. Test Procedures

**Figure 7:**
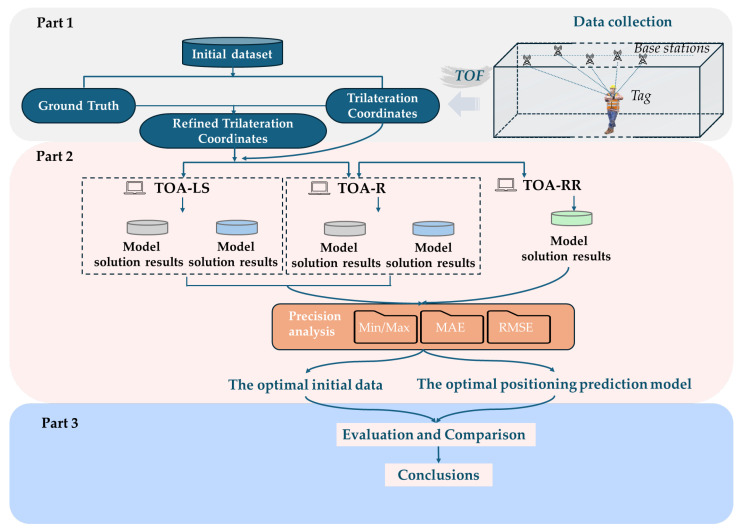
Test flowchart.

The experimenters established multiple measurement points at 60 cm intervals along the experimental corridor and used a total station to precisely measure their ground truth, providing a reference for comparing coordinates from UWB positioning models. They then wore tags to collect the UWB experimental data. To verify the accuracy and robustness of the TOA-R estimation model, one test scheme and three solution models were chosen. This process is depicted in the subsequent figure. See Figure 7.

① UWB base stations, labeled B1 through B5, are linearly arranged within the corridor. The experimenter dynamically traverses the predefined trajectory L, collecting signal data between the base stations and the tag under determination. The collected measurement data from the base stations and tags are transmitted to the control computer of the UWB positioning system in real time. These data serve as the raw data for subsequent offline coordinate calculations using the TOA model. Signal data collected within the first 5 s at the commencement and conclusion of the test are prone to interference and are consequently discarded.

② Following preprocessing, the TOA-LS model, TOA-R model, and TOA-RR model are utilized to process the data, and their coordinate solution accuracies are compared.

③ Root Mean Square Error (RMSE) is employed in the test to assess the positioning accuracy and the extent of environmental influence on the measurement data. Assuming there are *N* data points in the experiment, with the model solution coordinates being (*x_i_*, *y_i_*), and the actual coordinates being (${\hat{x}}_{i}$,${\hat{y}}_{i}$), then the RMSE is calculated as follows:

(28) $$RMSE=\sqrt{\frac{1}{N}\overset{N}{\underset{i=1}{\sum }}{\left({\hat{x}}_{i}-x_{i}\right)}^{2}+{\left({\hat{y}}_{i}-y_{i}\right)}^{2}}$$

## 4. Results and Discussion

### 4.1. Comparison of TOA-LS Model and TOA-R Model of Which the Trilateral Intersection Coordinates Are Used as the Initial Values

**Figure 8:**
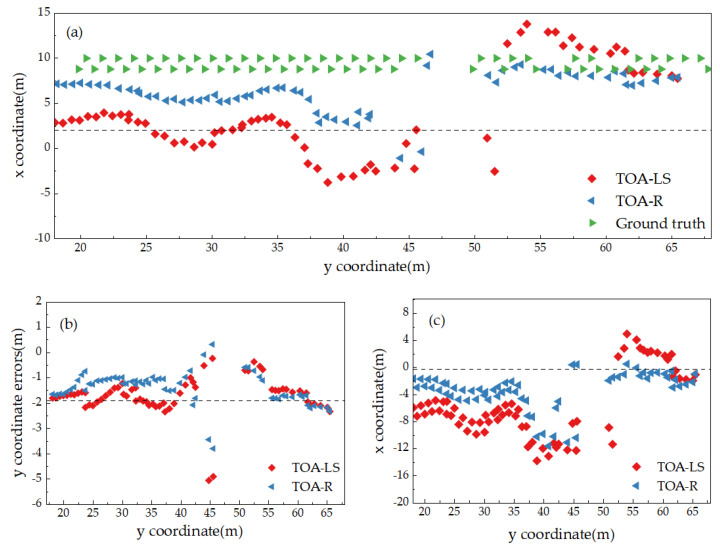
Comparison of positioning results between the TOA-LS model and TOA-R model: (**a**) measurement points, (**b**) *x*-coordinate errors, (**c**) *y*-coordinate errors.

Figure 8a presents the coordinate trajectory diagrams obtained by the TOA-LS model and the TOA-R model, both initialized with trilateration coordinates, as well as the actual point coordinate trajectory in the B1–B5 area. Figure 8b,c display comparison diagrams of positioning errors in the *y*-direction and *x*-direction between the positioning results of the TOA-LS and TOA-R models and the actual point coordinates. It can be observed from the figures that the TOA-R solution results are generally closer to the initial coordinate solution results, whereas the TOA-LS solution results exhibit larger local errors. Comparing Figure 8b,c, the positioning stability of both TOA-LS and TOA-R models is superior in the *Y*-axis direction compared to the *X*-axis direction. This aligns with the biased estimate characteristic of the TOA-R model, which has larger bias criteria by sacrificing less true errors.

Table 2 presents the error statistical results of the coordinates calculated by the TOA-LS and TOA-R models relative to the actual point position coordinates. The table lists the minimum absolute error (MinAE), maximum absolute error (MaxAE), mean absolute error (MAE), and root mean square error (RMSE) of the differences between the results obtained from the two models and the actual point coordinates. The coordinate difference values between the TOA-R model’s solution results and the actual point positions are as follows: in the *x*-direction, the average is 3.1715 m and the RMSE is 3.8568 m; in the *y*-direction, the average is 1.4000 m and the RMSE is 1.5255 m. Consequently, the calculation accuracy of the TOA-R model is superior to that of the TOA-LS algorithm. The results indicate that, due to the linear arrangement of the base stations along the *y*-axis, the *x*-coordinates of the base stations are highly correlated, leading to lower accuracy and poorer stability in the *x*-coordinate calculations by both models compared to the *y*-coordinate. Furthermore, when the initial coordinate error of the TOA-R model is significant, it can mitigate abnormal large errors and enhance the overall computational accuracy of the model.

### 4.2. Comparison of TOA-LS Model and TOA-R Model of Which the Refined Trilateral Intersection Coordinates Are Used as the Initial Values

**Figure 9:**
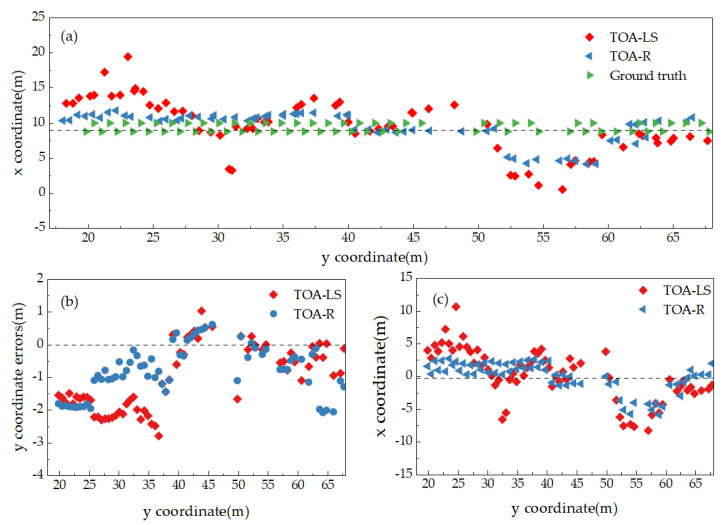
TOA-LS and TOA-R positioning with refined trilateral coordinates: (**a**) measurement points, (**b**) *x*-coordinate errors, (**c**) *y*-coordinate errors.

Figure 9a presents the coordinate trajectory diagrams obtained by the TOA-LS and TOA-R models, both initialized with refined trilateration coordinates, as well as the actual point coordinate trajectory in the B1–B5 region. Figure 9b,c, respectively, display the comparison diagrams of positioning errors in the *y*-direction and *x*-direction between the positioning results of the TOA-LS and TOA-R models and the actual point coordinates. It is evident from the figures that the TOA-R solution results are generally closer to the actual point positions, while the TOA-LS solution results exhibit high volatility. Figure 9b indicates that the errors of the TOA-LS and TOA-R solution results in the *y*-direction are slightly different from the errors of the actual point positions, which aligns with the effect that when the initial value is close to the true value, the operation is more stable and tends toward the true value. As shown in Figure 9c, the precision of the TOA-R solution in the *x*-direction is significantly better than that of the TOA-LS algorithm, which is consistent with the fact that TOA-R can reduce data collinearity and enhance the accuracy and robustness of the model solution, especially in cases of a strong linear correlation of data in the *x*-direction. A comparison with Figure 8 reveals that the use of refined initial values substantially reduces the *x*-coordinate localization error and, to a lesser extent, the *y*-coordinate error, depending on the consistency of the computational models.

Table 3 presents the error statistics between the TOA-LS and TOA-R model solution results, which utilize the refined trilateration coordinates as initial values, and the actual point coordinates. The table includes the MinAE, MaxAE, MAE, and RMSE of the differences between the results obtained from the two models and the actual point coordinates. For the TOA-R model versus the actual point coordinates, the average coordinate difference in the *x*-direction is 1.7764 m and the RMSE is 2.2697 m; in the *y*-direction, the average is 0.9104 m and the RMSE is 1.1040 m. The results indicate that the TOA-R model performs better than the TOA-LS model when initial values closer to the true values are used. Compared to the data in Table 2, when the TOA-LS and TOA-R models are solved with initial values closer to the true values, the solution accuracy is higher than when using trilateration coordinates as initial values, and the solution stability of the TOA-R model remains superior to that of the TOA-LS model.

### 4.3. Comparison of the TOA-RR Iterative Model

**Figure 10:**
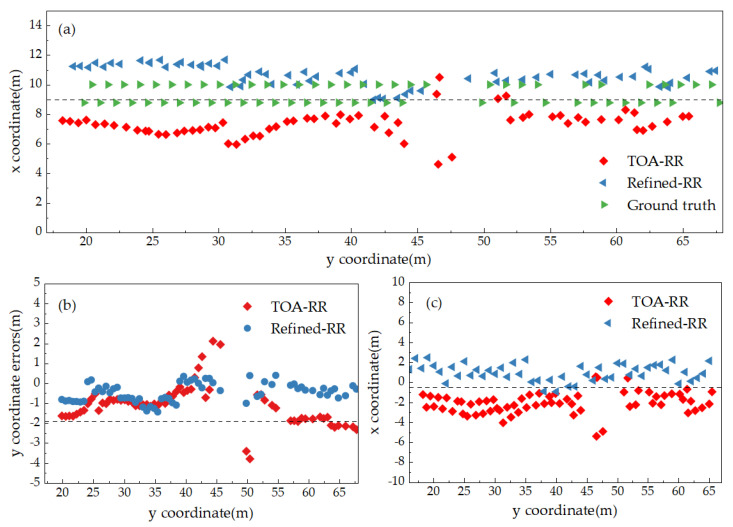
TOA-RR positioning with trilateral coordinates and refined trilateral coordinates: (**a**) measurement points, (**b**) *x*-coordinate errors, (**c**) *y*-coordinate errors.

Figure 10a presents the coordinate trajectory diagrams obtained by the TOA-RR model, initialized with coordinates computed by both the trilateration method and the refined trilateration intersection coordinates, as well as the actual point coordinates trajectory in the B1–B5 region. Figure 10b,c show the differences between the TOA-RR model positioning results, which are computed by using the trilateration intersection method and the refined trilateration solution coordinates as initial values, respectively, and the actual point position coordinates in the *Y*-axis and *X*-axis directions. As shown in Figure 10, the TOA-RR model solution, calculated with the refined trilateration coordinates as initial values, is found to be more closely aligned with the actual points in most cases. As indicated in Figure 10b,c, the iterative solution with the refined trilateral solution coordinates as the initial value provides a closer approximation to the actual point coordinates in the Y-direction. Compared to Figure 9, the results obtained with the TOA-RR model, when taking the refined trilateration coordinates as the initial value, are closer to the actual point coordinates, thus showing clear advantages over the TOA-R algorithm. This confirms that when the initial value is closer to the true value, a result closer to the true value can be achieved through multiple iterations of the calculation.

Table 4 presents the error statistics of the differences between the coordinates calculated by the TOA-RR model, which uses the coordinates computed by the trilateration intersection method and the refined coordinates as initial values, and the actual coordinates. As observed from the table, the coordinates obtained by the TOA-RR model with the refined trilateration solution coordinates as initial values fall within the error range of (0.0095 to 1.4357) meters in the *y*-direction, with the positioning accuracy for the *x*-coordinate ranging from (0.0543 to 2.8949) meters. The average error for the *x*-direction coordinate is 1.3241 m and the RMSE is 1.5347 m. For the *y*-coordinate, the average error is 0.5272 m and the RMSE is 0.6443 m. Consequently, the results obtained by the TOA-RR model solution, using the refined trilateration coordinates as initial values, exhibit greater accuracy, with more pronounced optimization in the positioning accuracy of the *x*-coordinate, and the overall solution accuracy surpasses that of other methods. A comparison of Table 2, Table 3, and Table 4 reveals that the overall resolution accuracy of the TOA-RR is superior, as the accuracy can be enhanced through iterative operations, particularly in the *x*-direction. However, due to the strong correlation of the *x*-direction data, the positioning accuracy of the *x*-coordinate remains lower than that of the *y*-coordinate.

**Figure 11:**
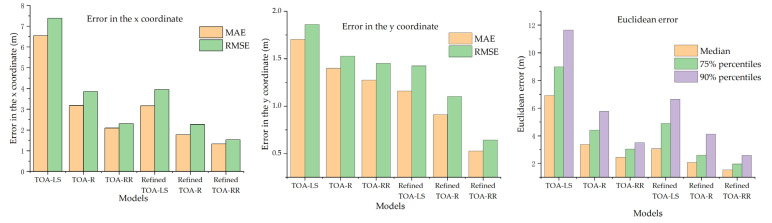
Comparison of positioning errors of each model.

The TOA-LS, TOA-R, and TOA-RR models, which use trilateration for initial positioning, and their refined counterparts (Refined TOA-LS, Refined TOA-R, and Refined TOA-RR) are compared in Table 5. This table lists their errors in the x and y directions, as well as the Euclidean errors. A comparative analysis of these errors reveals that the TOA-RR model outperforms the other two models in positioning accuracy. Moreover, after an initial value correction, the Refined TOA-RR model demonstrates the highest positioning accuracy. See Figure 11.

## 5. Conclusions

A novel UWB iterative Ridge estimation algorithm, termed TOA-RR, was developed to address the issue of linear correlation in base station positions within UWB positioning systems in long linear areas, such as corridors or underground tunnels. This algorithm addresses the distortion-prone solution issue inherent in traditional UWB solver models. The experimental tests led to the following conclusions.

The new TOA-RR algorithm model for UWB positioning is an iterative solution method that builds upon the TOA positioning method and Ridge estimation techniques. By incorporating a ridge parameter into the normal equation, the TOA-RR algorithm alters the complex multicollinearity of the original coefficients, resulting in enhanced positioning accuracy in long linear areas. The equivalent weight adjustment in the TOA-RR algorithm for UWB positioning is achieved through the iterative correction of observed values to compute equivalent weight factors, the method similar to the Huber function. It is an adaptive weight adjustment method. This approach effectively reduces the impact of positioning errors due to significant deviations in initial values during the linearization of the TOA equation and keeps the overall positioning error within a controlled range. The results from the dynamic pedestrian UWB positioning tests in long linear corridors indicate that when the initial value deviation of the undetermined tag is large or close to the true value, the TOA-LS model exhibits severe abnormal solutions, with calculated point coordinates deviating by several meters. In contrast, while the TOA-R model also shows significant coordinate errors, it maintains a stable solution. The proposed new TOA-RR algorithm has the potential to enhance the precision of the *y*-coordinate by approximately 0.5 m, significantly improving the positioning accuracy and providing a novel method to bolster the stability of UWB positioning.

Our study also includes a detailed description of the experimental design, data collection, and analysis methods. By comparing the TOA-RR algorithm with previous algorithms, we highlight its unique advantages and potential applications in solving UWB positioning problems in long linear areas. Future research could explore further improvements to the algorithm and its application in other environments.

The new TOA-RR algorithm model for UWB positioning is an iterative solution method that builds upon the TOA positioning method and Ridge estimation techniques. It incorporates a ridge parameter into the normal equation, altering the complex multicollinearity of the original normal equation’s coefficients and enhancing the positioning accuracy in long linear areas. The equivalent weight adjustment in the TOA-RR algorithm for UWB positioning is achieved through an iterative correction of observed values to compute equivalent weight factors akin to the Huber function, an adaptive weight adjustment method. This approach effectively reduces the impact of positioning errors due to significant deviations in initial values during the linearization of the TOA equation and keeps the overall positioning error within a controlled range. The results from the dynamic pedestrian UWB positioning tests in long linear corridors indicate that when the initial value deviation of the undetermined tag is large or close to the true value, the TOA-LS model exhibits severe abnormal solutions, with calculated point coordinates deviating by several meters. In contrast, while the TOA-R model also shows significant coordinate errors, it maintains a stable solution. The proposed new TOA-RR algorithm has the potential to enhance the precision of the *y*-coordinate by approximately 0.5 m, significantly improving the positioning accuracy and providing a novel method to bolster the stability of UWB positioning.

## Figures and Tables

**Table 1:** Coordinates of base stations in the test area.

Base Station Name	*X* Coordinate (m)	*Y* Coordinate (m)
B1	9.620	13.610
B2	9.620	37.560
B3	9.620	72.770
B4	7.896	24.984
B5	7.971	55.404

**Table 2:** TOA-LS and TOA-R positioning accuracy 1.

Model	Error in the *x* Coordinate (m)				Error in the *y* Coordinate (m)			
	MinAE	MaxAE	MAE	RMSE	MinAE	MaxAE	MAE	RMSE
TOA-LS	0.4341	13.7390	6.5375	7.3834	0.2231	5.0378	1.7043	1.8619
TOA-R	0.0330	11.0531	3.1715	3.8568	0.0779	3.7878	1.4000	1.5255

**Table 3:** TOA-LS and TOA-R positioning accuracy 2.

Model	Error in the x Coordinate (m)				Error in the y Coordinate (m)			
	MinAE	MaxAE	MAE	RMSE	MinAE	MaxAE	MAE	RMSE
TOA-LS	0.0678	10.6881	3.1626	3.9490	0.0080	2.7858	1.1605	1.4242
TOA-R	0.0152	5.8418	1.7764	2.2697	0.0461	2.0778	0.9104	1.1040

**Table 4:** TOA-RR positioning accuracy.

Model	Error in the x Coordinate (m)				Error in the y Coordinate (m)			
	MinAE	MaxAE	MAE	RMSE	MinAE	MaxAE	MAE	RMSE
TOA-RR	0.4676	5.3673	2.0905	2.3047	0.1736	3.7820	1.2739	1.4529
Refined-TOA-RR	0.0543	2.8949	1.3241	1.5347	0.0095	1.4357	0.5272	0.6443

**Table 5:** Summary of errors of each positioning model.

Model	Error in the x Coordinate (m)		Error in the y Coordinate (m)		Euclidean Error (m)		
	MAE	RMSE	MAE	RMSE	Median	75% Percentiles	90% Percentiles
TOA-LS	6.5375	7.3834	1.7043	1.8619	6.9069	8.9860	11.6416
TOA-R	3.1715	3.8568	1.4000	1.5255	3.3675	4.4076	5.7558
TOA-RR	2.0905	2.3047	1.2739	1.4529	2.4599	3.0460	3.5038
Refined TOA-LS	3.1626	3.949	1.1605	1.4242	3.0694	4.8854	6.6519
Refined TOA-R	1.7764	2.2697	0.9104	1.104	2.0719	2.6008	4.1243
Refined TOA-RR	1.3241	1.5347	0.5272	0.6443	1.5373	1.9557	2.5864

## Data Availability

The original contributions presented in the study are included in the article; further inquiries can be directed to the corresponding authors.
